# The Mediation Effect of Self–Report Physical Activity Patterns in the Relationship between Educational Level and Cognitive Impairment in Elderly: A Cross-Sectional Analysis of Chilean Health National Survey 2016–2017

**DOI:** 10.3390/ijerph17082619

**Published:** 2020-04-11

**Authors:** Patricio Solis-Urra, Julio Plaza-Diaz, Ana Isabel Álvarez-Mercado, Fernando Rodríguez-Rodríguez, Carlos Cristi-Montero, Juan Pablo Zavala-Crichton, Jorge Olivares-Arancibia, Javier Sanchez-Martinez, Francisco Abadía-Molina

**Affiliations:** 1 PROFITH “PROmoting FITness and Health through Physical Activity” Research Group, Department of Physical Education and Sport, Faculty of Sport Sciences, University of Granada, 18071 Granada, Spain; 2 IRyS Research Group, School of Physical Education, Pontificia Universidad Católica de Valparaíso, Valparaiso 2374631, Chile; fernando.rodriguez@pucv.cl (F.R.-R.); carlos.cristi.montero@gmail.com (C.C.-M.); jorge.olivares.ar@gmail.com (J.O.-A.); 3 Department of Biochemistry and Molecular Biology II, School of Pharmacy, Campus de Cartuja s/n, University of Granada, 18071 Granada, Spain; jrplaza@ugr.es (J.P.-D.); alvarezmercado@ugr.es (A.I.Á.-M.); 4 Institute of Nutrition and Food Technology “José Mataix”, Biomedical Research Center, Parque Tecnológico Ciencias de la Salud, University of Granada, 18016 Granada, Spain; 5 Instituto de Investigación Biosanitaria ibs. GRANADA, 18014 Granada, Spain; 6 Faculty of Education and Social Sciences, Universidad Andrés Bello, Viña del Mar 2531015, Chile; jzavala@unab.cl; 7 Escuela de Pedagogía en Educación Física, Facultad de Educación, Universidad de las Américas, Santiago 8370035, Chile; 8 Escuela de Kinesiología, Facultad de Salud, Universidad Santo Tomás, Viña del Mar 2561694, Chile; 9 Department of Cell Biology, School of Sciences, University of Granada, 18071 Granada, Spain

**Keywords:** cognitive function, aging, sedentary behavior, exercise, mental health

## Abstract

The aims of this cross-sectional study were (i) to determine the association of educational level attained with cognitive impairment and (ii) to investigate the mediating effect of different self-report physical activity (PA) patterns in a large sample of older Chileans. A sample of 1571 older adults from the National Chilean Survey (2016–2017) was included. The educational level attained, PA levels, mode of commuting, sedentary time, and leisure-time PA were self-reported through validated questionnaires. Cognitive impairment was determined by Mini-Mental State Examination (modified version). Association between educational level attained and cognitive impairment was examined using logistic regression models. Counterfactual mediation models were used to test the mediating effect of self-reported PA patterns. A lower educational level was consistently associated with higher odds of cognitive impairment (OR range 2.846 to 2.266, all *p* < 0.001), while leisure-time PA was the only PA pattern that partially mediated this association (proportion mediated 8.0%). In conclusion, leisure-time PA was the solely PA pattern that partially mediated the association between the educational level and cognitive impairment. The rest self-reported PA patterns did not modify this association.

## 1. Introduction

Nearly 10 million new cases of dementia are diagnosed every year, with an expectation of a three-fold increase by 2050 [1,2]. Dementia and some grades of cognitive impairment have several consequences, such as increasing disability and reduced quality of life, for both patients and caregivers. Besides this, dementia significantly interferes with the maintenance of daily living activities, being one of the principal causes of dependence among older adults [1]. As a consequence, the prevention of neurocognitive disorders such as dementia or cognitive impairment is a public health priority worldwide [1,3]. Nowadays it is vital to give more emphasis on several modifiable risk factors, such as education attainments and physical activity (PA), among others [2].

Education is an important social factor that leads to human development and economic growth [4] as well as being a powerful predictor of future mental health [5]. Continuing education has been associated with better cognitive function and lower incidence of dementia [5]. For example, it was found that a higher educational level reduced the possibility of risk of cognitive impairment during aging, protecting against the decline of executive and global function [6]. 

On the other hand, PA is the fourth biggest factor worldwide related to global mortality, and it is one of the main modifiable risk factors [7]. PA has been favorably associated with several health outcomes such as vascular and metabolic disease [8], frailty [9], and others. Emerging evidence suggests that PA is linked to cognitive function [10], thereby it presents a vital and protective role in mental health [11]. Older adults reporting higher levels of PA have fewer grades of depression, cognitive impairment, and even lower incidence of dementia [11]. Different domains of PA, which include leisure-time, active commuting as well as the global level of PA and sedentary time, have been associated differently with cognitive impairment and mental health outcomes. For example, lower levels of leisure-time have been associated with greater cognitive decline [12]. Additionally, higher levels of global PA have been inversely associated with the risk of dementia [10]. Interestingly, a recent work indicated that there was a 76.1% consensus that PA had positive cognitive effects [13].

Changes in physiological, pathological, social, and psychological conditions are associated with aging [14]. The World Population Prospects 2019 indicates that by 2050, one in six people around the world will be over the age of 65 [15,16]. Likewise, both medical advances and improvements in lifestyle profoundly influence longevity. Then, longevity should be accompanied by healthy aging. This tremendous social challenge is fundamental due to life expectancy increasing faster than the period of life in good health for the elderly, according to the challenges of the European Union termed “healthy life years” [8,17]. For this reason, there is a lot of interest in how modifiable risk factors can play a role in attenuating or increasing the probability of cognitive impairment.

In this regard, there is extensive evidence suggesting the independent contribution of educational level as well as PA in relation to cognitive functioning. However, there is a lack of an approach combining both factors. In this sense, a mediation analysis could provide valuable information about this insight, as it provides evidence of the different association and pathways between sets of variables [18]. Thus, as attained educational level is known as a factor associated with cognitive impairment, it is interesting to evaluate which modifiable risk factors might mediate the pathway of these variables. In this line, recent work found that physical fitness (a proxy report of PA) partially mediated the association between the educational level of the family and academic achievement, suggesting the relevance of PA as a mediating factor [18]. According to our knowledge, there is no previous study that tests the mediation effect of different PA domains on the association between attained educational level and cognitive impairment in older adults, which is important, as PA patterns have shown to play a key role mediating associations between lifestyles factors and several health conditions [19,20].

Therefore as mentioned above, attained educational level is known as a predictive factor related to cognitive impairment, it seems to be interesting to evaluate whether PA patterns (global PA level, leisure-time PA, commuting mode, and sedentary time) might significantly mediate this association. Accordingly, this study aims (i) to determine the association of educational level attained with cognitive impairment adjusted for several covariates and (ii) to investigate the mediating effect of different self-report PA patterns in this association in a large sample of older Chileans.

## 2. Materials and Methods

### 2.1. Study Population

The 2016–2017 Chilean National Health Survey is a representative household survey with a stratified multistage probability sample of 6233 non-institutionalized participants over 14 years old from the 15 regions of Chile, both urban and rural. The sample size was calculated with a relative sampling error of less than 30%, and an absolute sampling error of 2.6% to the national level. The participation rate was 90.2%. Detailed information about the survey has been described elsewhere [21]. In this study, we evaluated 1671 older adults (>60 years). Of these respondents, 1571 had recorded values of PA, cognitive impairment, educational level as well as covariates included. The ethics committee of the Pontificia Universidad Católica de Chile and the Chilean Ministry of Health approved the study protocol and ethical consent forms.

### 2.2. Survey and Sample

Standardized protocols were used, and all investigators (nurses and research technicians) underwent joint training sessions before the implementation of the survey. The time framework for this survey was conducted between August 2016 and March 2017.

### 2.3. Educational Level

Educational level was established according to information of the highest year of education reached reported by the participant. The response was registered in years and then categorized in (i) less than 8 years (primary); (ii) between 8 and 12 years (secondary) and (iii) 13 years or beyond (beyond secondary). Then, due to very few cases in the highest level of education (beyond secondary), this variable was dichotomized and used as primary, and beyond the primary educational level.

### 2.4. Physical Activity Patterns

#### 2.4.1. Global Level of Physical Activity

The Global Physical Activity Questionnaire (GPAQ) (version 2) was used to measure PA. The categories of PA were defined according to the standard criteria of the questionnaire. Those who had less than 600 metabolic equivalents of task (METS) per week were considered inactive and those who had 600 or more METS per week were considered active [22].

#### 2.4.2. Leisure-Time Physical Activity

These questions were performed in the visit to participant’s homes: In the last month, did you practice a sport or do any physical activity out of work time, during 30 min or more each time? The response options were: (i) Yes, three times a week or more; (ii) Yes, one or two times a week; (iii) Yes, less of four times per month; (iv) I do not practice a sport or physical activity. Then the responses were categorized as “no” for those who did not practice a sport or PA, or as “Yes” for those who declared any practice of sport or leisure-time PA.

#### 2.4.3. Commute Mode

To inquire about the commute mode of those surveyed it was asked: Which is the mode of commuting that you use at least one time per week? The response options were: (i) drive a light car; (ii) drive a heavy car; (iii) light car passenger; (iv) heavy car passenger; (v) bicycle; (vi) walk; and (vii) other. The responses were categorized as the mode of “active commuting” for those who used a bicycle or walked, or as “passive commuting” for the rest of the modes.

#### 2.4.4. Sedentary Time

A question of the GPAQ to estimate sedentary time was asked. The question was (i) *How much time do you usually spend sitting or reclining on a typical day?* The participant had to respond in minutes and hours per day. This question was categorized according to non-sedentary (<4 h per day) and sedentary (≥4 h per day) [23], which has been previously used in this population [24]. 

### 2.5. Cognitive Function

The modified Mini-Mental State Examination (mMMSE) was used to determine cognitive impairment [25]. This instrument was derived from a previously validated version of the MMSE in the Chilean population [26]. This method has been previously used to determine cognitive impairment in the Chilean population, and was used to identify cognitive impairment in the 2016–2017 Chilean national survey [24,26,27]. Briefly, the mMMSE comprises different questions with a maximum total score of 19 points. The categories of response were yes or no, according to different domains corresponding to spatial orientation, relation between objects, verbal fluency, and memory (i.e., date and place of birth, word fluency, similarities, and delayed recall of words). Respondents with scores 13 or less were considered cognitively impaired [24,26,27,28]. The complete instrument and specifications can be found in an open-access link from the website of the Chilean Ministry of Health (http://epi.minsal.cl/encuesta-nacional-de-salud-2015-2016/). A set of sociodemographic and health variables were collected through face-to-face household interviews [28].

### 2.6. Covariates

A set of possible covariates was selected to include in the models. The covariates were: age in years, gender, and body mass index computed as weight in kilograms divided by height in meters squared (kg/m^2^); a general question of well-being: *How do you feel with your life in general (with your work, family, well-being, health, love)?* The range of response was 1–5, 5 being a great feeling and 1, a poor feeling. Furthermore, a composed healthy diet index was used as a covariate, which includes (i) >1 serving/day, grain (1 point) or less (0 points); (ii) >1 serving/week fish (1 point) or less (0 points), (iii) ≤4.5 servings/week sweetened beverages (1 point) or more (0 points) and iv) ≥4.5 servings/day fruits and vegetables (1 point) or less (0 points); thus, a score between 0 to 4 was created, 4 being a great score and 0, a poor score of a healthy diet [29]. Finally, a question about depression treatment: *Have you ever received depression treatment?* Responses were yes/no.

### 2.7. Statistical Analysis

Data are presented as mean, standard deviation (SD) for continuous variables, and as absolute (*n*) and relative (%) frequencies for categorical variables. Independent t-test and chi-square tests were used to compare differences between participants with and without cognitive impairment for continuous and categorical variables, respectively. Firstly, logistic regression was performed to estimate the odds ratio (OR) and 95% CI to establish the association between educational level and cognitive impairment using the highest level as reference (beyond primary). Logistic regression models were used to provide more valid estimates of the incidence density ratio in cross-sectional analyses [30]. Four models are shown: an unadjusted model (model 1); an model adjusted by age and sex (model 2); model 2 plus body mass index and self-reported well-being (1–5) (model 3); and model 3 plus self-reported depression treatment (yes/no) and a healthy diet index (0–4) (model 4). Then, we used Akaike’s Information Criterion (AIC) to compare the candidate model to include in the counterfactual mediation analysis. The model with the lowest AIC was selected as the best of the candidate models and was included in the mediation analysis. 

**Figure 1:**
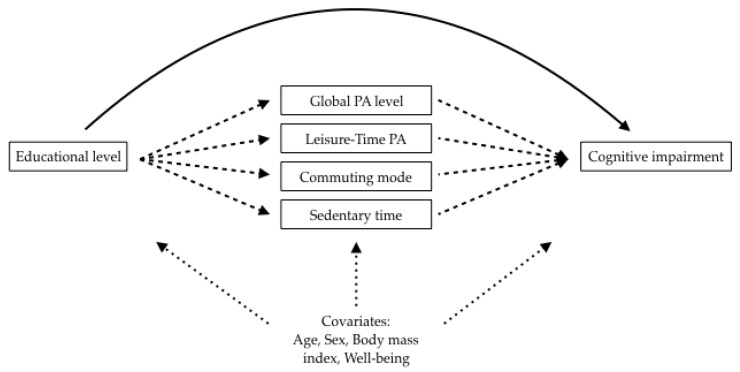
Causal diagram of the mediation hypothesis. The solid arrow line displays the natural direct effect pathway and the arrows with dashed lines display the natural indirect pathway. The three-pointed arrow lines display the confounding variables. PA: physical activity.

Then, we performed counterfactual mediation analyses to quantify the amount of cognitive impairment, explained by each mediating PA pattern, using the educational levels as an explicative variable (see Figure 1). In order to get simple interpretation, as mentioned above, each PA pattern also was dichotomized, thus, the global level of PA corresponding to active/inactive, leisure-time PA corresponding to yes/no, commute mode corresponding to active/passive, and sedentary time corresponding to non-sedentary/sedentary. Then, we obtained the natural indirect effects (NIE), which denote the part of the total effect of the educational level that is mediated by each PA pattern. While natural direct effects (NDE) reflect the rest of the total effect [31,32]. Therefore, the total effect represents the change in OR observed when all older adult’s educational levels are changed from the reference level (beyond primary) to the other level of educational level (primary). NDE represents the change in OR when all older adult’s educational levels are changed from the reference level to the other educational level while fixing the older adult’s PA pattern to the level it naturally takes at the reference level of educational level. Lastly, NIE represents the OR observed when all older adult’s PA patterns are changed from the value they naturally take at the reference level (beyond primary), to the value they take naturally at the new educational level, while keeping the educational level fixed at the reference level. All mediation analyses were adjusted by covariates included in model 3. The proportion mediated through each PA mediator was calculated on the risk difference scale. The proportion mediated was calculated as [ORNDE (ORNIE − 1)]/[ORNDE × ORNIE − 1] × 100%, for the binary outcome [33]. All analyses were performed using R version 3.6.1 (R Foundation for Statistical Computing, Vienna, Austria), and package “medflex” was used for nested counterfactuals mediation analysis with an imputation approach [31,32,34]. A value of *p* < 0.05 was considered statistically significant.

## 3. Results

### 3.1. Demographic Analysis of the Study Population

Table 1 shows the descriptive characteristics of the participants. The initial sample was in 1670 participants, corresponding to valid data of educational levels, covariates, and cognitive impairment variables. The final sample was divided into adults with cognitive impairment (<13 mMMSE) and without cognitive impairment (≥13 mMMSE). Then, the final sample with complete data of PA patterns to include in the mediation was 1571. 

Statistical differences between groups were observed in age, body mass index, and well-being. The cognitive impairment group had the oldest participants with less body mass index and higher well-being compared with the group without impairment. Healthy diet index, sex distribution, and depression treatment were similar in both groups. Educational level and PA variables were significantly different between groups except for the mode of commuting and sedentary time groups (Table 1).

### 3.2. Association between Education Level and Cognitive Impairment

Results of the four models on the relationship between educational level attained and cognitive impairment are presented in Table 2. All of the models show a consistent association between the educational level attained and cognitive impairment. The lower educational level attained (primary) was associated positively with the ODDs of cognitive impairment (OR range 2.846 to 2.266, all *p* < 0.001). Finally, the model with the lowest AIC was selected as the best of the candidate models and was included in the mediation analysis (model 3).

### 3.3. Mediation Analyses Results

**Figure 2:**
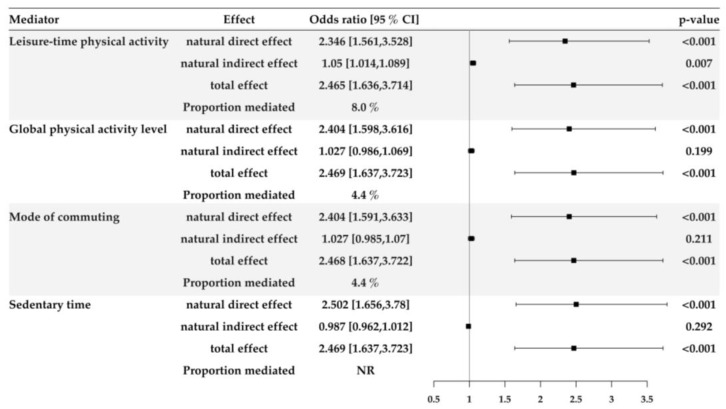
Counterfactual mediation analysis on primary vs beyond primary (reference) educational level. NR is expressed when the natural direct and natural indirect relationships were not in the same direction; therefore, the mediated proportion is not a logical value.

Results from the mediation analyses are displayed in Figure 2. Mediation analyses were performed to quantify the amount of cognitive impairment, explained by each mediating PA pattern, using educational levels as an explicative variable. The Leisure-time PA was the sole PA pattern that partially mediated the association with 8.0% of the mediated proportion. Both the global PA levels as the mode of commuting show 4.4% of proportion mediated, although it did not reach statistical significance.

## 4. Discussion

The main findings of the present study supported the association between the attained educational level and cognitive impairment. Additionally, the counterfactual mediation analysis showed that PA patterns have a minimal mediation effect in the above-mentioned relationship, suggesting that the effect is principally provided partially by leisure-time PA. These results suggest that a self-reported PA pattern is not a complete pathway by which the attained educational level is associated with cognitive impairment, suggesting that other modifiable or non-modifiable factors might affect this association. A detailed explanation of these points will be provided in the following sections.

To our knowledge, this is the first study that analyzes different PA patterns as the pathway of the association between attained educational levels and cognitive impairment in older adults. Our finding associating the educational level and cognitive impairment was similar to that of Moreno et al. in a previous analysis with the same data [28]. The association of the PA pattern with cognitive impairment has been examined in a similar large-scale data among older Chilean adults [24] as well as several other modifiable and non-modifiable factors linked with cognitive impairment [35]. Of note in our study is the association of cognitive impairment with the early stages of dementia. Paying attention to older adults with lower educational levels as well as low physical activity might be important during aging, especially because both factors have been associated with the prevalence of dementia and risk of neurodegenerative diseases [36]. In this study, we proposed PA patterns as a pathway by which the attained educational level is related to cognitive impairment. This hypothesis is supported by several previous evidence that indicates how both low attained educational levels and low PA can be associated or even can be lead to cognitive impairment in adults [11]. In this line, while leisure-time PA shows a slight partial mediation effect, no other self-reported PA patterns showed a mediating effect.

Even though PA patterns have been consistently associated with physical health, our findings provide an unclear role on a mental health outcome such as cognitive impairment in older adults. A previous work found that self-reported PA was consistently associated with an increased risk of incident diabetes, coronary heart disease, and stroke, but not with the incidence of dementia [37]. According to different PA patterns, two studies have reported a positive association between leisure-time PA and cognition in older adults but failed to find an association with commuting mode [38,39]. About the association between sedentary time and cognitive outcomes, a recent systematic review showed that there is a lack of association between these variables in older ages [40]. However, different types of sedentary behaviors may have diverse effects on cognitive outcomes, so future research to ask this question should be performed. [41].

Another possibility that might explain our results is reverse causality. In support of this, a recent work revealed that the association of PA patterns and cognitive impairment could be due to reverse causality. [37] This suggests that cognitive impairment could lead to lower levels of physical activity and not in an inverse manner, as shown in the decline in physical activity levels in the early stages of dementia [42]. In this line, a mendelian randomization approach used in a recent study supports this hypothesis in patients with Alzheimer’s disease, mentioning that previous findings of observational studies might have been biased [43]. Consequently, our results are somewhat expected, where PA patterns seem to not mediate the pathway between attained educational level and cognitive impairment. It is important to note that the PA patterns derived from a self-report should be interpreted with caution, at least in this specific scenario. With regard to this, it has been described that educational level is related to the level of validation in self-report PA questionnaires. Lower educated populations have less validation in this questionnaire [44] due to the over-estimation of PA levels compared to people with higher educational levels. [45]. 

For this reason, it has been recommended that self-reported PA patterns should be interpreted with caution in older adults, as the cognitive function is a factor that could bias subjective PA patterns [46].

Finally, the PA patterns were measure with the GPAQ, sum to two simple questions about leisure-time PA and commute mode. Despite that this questionnaire is the most used worldwide, a recent systematic-review has mentioned that no research has specifically examined the reliability and validity of the GPAQ in older adults, as well as in American countries [47]. Additionally, the GPAQ do not have questions about specific spaces of PA nor social interaction of PA, which may be related to cognitive impairment [48]. Another important aspect not considered in this instrument is light PA, that has been associated with cognitive decline independently to moderate PA [48]. 

Important strengths of the present work include the population-based sampling method and the wide consideration of potential confounders. Additionally, we examined the NIE of several potential PA mediators of the association of interest, such as the global level of PA, leisure-time PA, commute mode, and sedentary time. The principal limitation in our study is that the cross-sectional design does not allow us to draw causal relationships, as was addressed above. As was mentioned, PA patterns were self-reported; thus, significant bias and pitfalls emerge when the instrument is applied to people with different educational levels and older populations, as well as in the relationships with cognitive outcomes. Another relevant problem is the lack of measurement of light intensity PA, which is an essential PA component, especially in older adults. Both difficulties mentioned could be solved with an objective measurement of PA, such as an accelerometer or pedometer that have been used in the elderly, or by including questions about types of PA in specific contexts. Hence, the limitations of this study may lead to an underestimation of the mediation effect of PA on the association between educational levels and cognitive impairment. In light of solving these pitfalls, more research is needed to confirm or refute these results.

## 5. Conclusions

In conclusion, our results support previous association between attained educational levels and cognitive impairment in a large sample of older Chilean adults. Additionally, while the leisure-time PA showed a slight partial mediation effect, the remaining self-reported PA patterns did not present a mediation effect. These results provide an understanding of how lifestyle factors, such as self-report PA patterns, could mediate the association between attained educational level and cognitive impairment. Future research should focus on identifying whether PA patterns is a pathway by which attained educational level is associated with cognitive impairment solving, considering the issues mentioned above.

## Figures and Tables

**Table 1:** Descriptive characteristic of elderly adults according to cognitive status.

	Cognitive Impairment (<13 mMMSE) *n* = 178	Without Cognitive Impairment (≥13 mMMSE) *n* = 1492	*p*-Value
	Mean ± SD	Mean ± SD	
Age (years)	75.3 ± 9.2	70.2 ± 7.4	<0.001
Body mass index (kg/m^2^)	28 ± 4.9	29.3 ± 5.3	<0.001
Well-being	2.8 ± 0.8	2.7 ± 0.7	0.02
Healthy diet index	1.8 ± 0.6	1.8 ± 0.6	0.84
Sex	*n* (%)	*n* (%)	
Male	73 (41)	528 (35.4)	0.163
Female	105 (59)	964 (64.6)	
Depression treatment			
Yes	30 (16.9)	305 (20.4)	0.303
No	148 (83.1)	1187 (79.6)	
Educational level			
Primary	129 (72.5)	717 (48.1)	<0.001
Beyond primary	49 (27.5)	775 (51.9)	
Global physical activity level *			
Active	49 (32.2)	764 (53.8)	<0.001
Inactive	103 (67.8)	655 (46.2)	
Leisure-Time physical activity *			
Yes	4 (2.6)	162 (11.4)	0.01
No	148 (97.4)	1257 (88.6)	
Mode of commuting *			
Active commuting	46 (30.3)	338 (23.8)	0.09
Passive commuting	106 (69.7)	1081 (76.2)	
Sedentary time *			
Sedentary	131 (86.2)	1158 (81.6)	0.198
Non-sedentary	21 (13.8)	261 (28.4)	

* As PA variables presented less valid cases, the final sample in these variables was in the 152 cognitive impairment group and 1419 in the group without cognitive impairment. Variables are presented as mean ± standard deviation (SD) for continuous scale and as absolute (*n*) and relative (%) frequencies for non-continuous scales. The *p*-value corresponds to the t-test for continuous and chi-square for categorical variables. mMMSE: modified Mini-Mental State Examination.

**Table 2:** The odds ratio for cognitive impairment according to different models.

Explanatory Variable	Model 1		Model 2		Model 3		Model 4	
	OR [95% CI]	*p*-Value	OR [95% CI]	*p*-Value	OR [95% CI]	*p*-Value	OR [95% CI]	*p*-Value
Educational level								
Beyond primary (ref.)	1		1		1		1	
Primary	2.846 [2.03,4.048]	<0.01	2.266 [1.593,3.265]	<0.01	2.305 [1.611,3.341]	<0.01	2.32 [1.62,3.366]	<0.01
Age			1.07 [1.049,1.091]	<0.01	1.064 [1.042,1.085]	<0.01	1.064 [1.042,1.086]	<0.01
Sex (female)			0.704 [0.507,0.981]	0.037	0.737 [0.529,1.03]	0.072	0.734 [0.523,1.034]	0.075
Well-being					1.195 [0.966,1.476]	0.1	1.199 [0.968,1.485]	0.096
Body mass index					0.96 [0.927,0.994]	0.022	0.96 [0.927,0.993]	0.021
Healthy diet index							1.129 [0.853,1.494]	0.394
Depressive treatment (No)							0.952 [0.605,1.458]	0.826
Goodness of fit								
AIC	1.098.085		1.053.011		1.049.208		1.052.428	

OR: Odds ratio; 95% CI: 95% confidence interval; AIC: Akaike information criterion; In order to identify the model to include in the counterfactual mediation analysis, the most parsimonious model is selected according to the lowest AIC (Model 3).
